# Bioinformatics Analysis of the Lipoxygenase Gene Family in Radish (*Raphanus sativus*) and Functional Characterization in Response to Abiotic and Biotic Stresses

**DOI:** 10.3390/ijms20236095

**Published:** 2019-12-03

**Authors:** Jinglei Wang, Tianhua Hu, Wuhong Wang, Haijiao Hu, Qingzhen Wei, Xiaochun Wei, Chonglai Bao

**Affiliations:** 1 Institute of Vegetables Research, Zhejiang Academy of Agricultural Sciences, Hangzhou 310021, China; syauwjl@163.com (J.W.); hutianh@126.com (T.H.); hongge5@163.com (W.W.); huhj0571@126.com (H.H.); weiqz@mail.zaas.ac.cn (Q.W.); 2 Institute of Horticulture, Henan Academy of Agricultural Sciences, Zhengzhou 450002, China; jweixiaochun@126.com

**Keywords:** radish, lipoxygenase (LOX), gene family, abiotic stress, tandem duplication

## Abstract

Lipoxygenases (LOXs) are non-heme iron-containing dioxygenases involved in many developmental and stress-responsive processes in plants. However, little is known about the radish LOX gene family members and their functions in response to biotic and abiotic stresses. In this study, we completed a genome-wide analysis and expression profiling of *RsLOX* genes under abiotic and biotic stress conditions. We identified 11 *RsLOX* genes, which encoded conserved domains, and classified them in 9-LOX and 13-LOX categories according to their phylogenetic relationships. The characteristic structural features of 9-LOX and 13-LOX genes and the encoded protein domains as well as their evolution are presented herein. A qRT-PCR analysis of *RsLOX* expression levels in the roots under simulated drought, salinity, heat, and cold stresses, as well as in response to a *Plasmodiophora brassicae* infection, revealed three tandem-clustered *RsLOX* genes that are involved in responses to various environmental stresses via the jasmonic acid pathway. Our findings provide insights into the evolution and potential biological roles of RsLOXs related to the adaptation of radish to stress conditions.

## 1. Introduction

Lipoxygenases (EC 1.13.11.12; LOXs), which are ubiquitously distributed in plants, animals, and fungi, are important monomeric, non-heme iron-containing dioxygenases [1] that catalyze the oxygenation of polyunsaturated fatty acids to produce fatty acid hydroperoxides [2]. A typical LOX comprises the following two major domains: a C-terminal LOX domain and an N-terminal polycystin-1, lipoxygenase, alpha-toxin (PLAT) domain [3]. The LOX domain contains five conserved histidine (His) residues that form a fragment [His-(X)4-His-(X)4-His-(X)17-His-(X)8-His] involved in the binding of an iron atom [4]. The PLAT domain, which contains an eight-stranded antiparallel β-barrel, can bind to procolipase to mediate membrane associations [5,6]. Depending on whether the oxidation of polyunsaturated fatty acids occurs at carbon atom 9 or at carbon atom 13, the plant LOXs are grouped in the 9-LOX or 13-LOX categories [2]. Additionally, LOXs may also be categorized as type I or type II based on their structural and sequence similarities. The type I LOX sequences are highly similar and do not encode a transit peptide, whereas the type II LOX sequences are only moderately similar and encode an N-terminal chloroplast transit peptide [7].

A previous study revealed that 9S-hydroperoxyoctadecadienoic acid (9-HPOD) and 13S-hydroperoxyoctadecadienoic acid (13-HPOD), which are the products generated by 9-LOX and 13-LOX, respectively, can be further converted to various oxylipins by a variety of enzymes [8]. In plants, LOXs and the derived oxylipins are reportedly involved in several biological events, including seed germination [9]; tuber development [10]; fruit ripening [11]; sex determination [12]; and, most importantly, immune responses [13,14,15].

The oxylipins derived from 13-HPOD, such as jasmonic acid (JA) and green leaf volatiles, are important for responses to abiotic and biotic stresses [16,17]. In maize, the 9-HPOD-derived products include death acids, such as 10-oxo-11-phytodienoic acid and 10-oxo-11-phytoenoic acid, which accumulate to inhibit infections by fungi and herbivorous insects when plants are infected with the pathogen responsible for southern leaf blight (*Cochliobolus heterostrophus*) [18,19]. Moreover, LOX activities have been detected during responses to biotic and abiotic stresses in diverse plant species. For example, six LOXs were identified in *A. thaliana* [20], with the *AtLOX1* and *AtLOX5* expression induced by stress-related hormones influencing immune responses [21,22] and *AtLOX2* and *AtLOX6* expression contributing to JA biosynthesis [23,24]. Among solanaceous plant species, tomato *SlLOXD* [15], tobacco *NaLOX3* [25,26], and potato *StLOXH3* [27] are involved in JA biosynthesis, whereas *NaLOX2* and *StLOXH1* mediate the biosynthesis of green leaf volatiles [28,29]. Furthermore, pepper *CaLOX1* contributes to the scavenging of reactive oxygen species and the activation of defense-related genes in responses to abiotic stresses [30]. In persimmon, *DkLOX3* plays a crucial role in the regulation of senescence and the tolerance to salt stress [31].

Several studies have investigated LOX genes using bioinformatic tools to clarify their functions in various physiological and molecular events in diverse plant species, including *Arabidopsis thaliana* [32], rice [20], cotton [33], maize [34], and cucumber [35]. Radish (*Raphanus sativus* L., 2*n* = 2*x* = 18), which belongs to the family Brassicaceae and a relative of *Brassica rapa* and *Brassica oleracea*, is a root vegetable cultivated worldwide. The growth and economic value of *R. sativus* are affected by a variety of biotic and abiotic stresses, such as extreme weather, saline–alkaline conditions, and diseases due to insect infestations. Because of the importance of the LOX gene family in responses to biotic and abiotic stresses, a comprehensive investigation of the *R. sativus* LOX genes is needed.

The present study involved a thorough analysis of the *R. sativus* LOX gene family. Specifically, 11 LOX genes were identified in the *R. sativus* genome based on bioinformatics resources and then characterized regarding the encoded enzyme sequences, chromosomal distribution, phylogenetic relationships, classifications, structure, encoded conserved motifs, synteny, tandem duplications, as well as expression patterns in various organs and in response to abiotic and biotic stresses. The results of our study may form the basis of future investigations aimed at more thoroughly characterizing the LOX genes in *R. sativus* and related species.

## 2. Results

### 2.1. Identification and Characterization of LOX Gene Family Members

**Figure 1:**
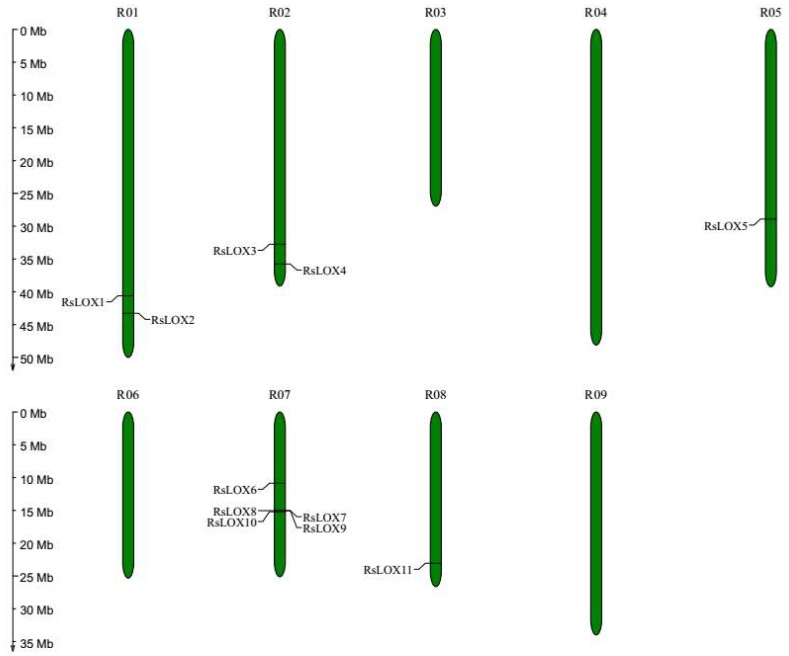
Distribution of *RsLOX* genes on *R. sativus* chromosomes. The line on the green bars indicates the location of LOX genes on chromosomes. The left values corresponding to the scales indicate chromosomes physical distance.

We applied a bioinformatics-based approach to identify 11, 14, 11, and six LOX genes in *R. sativus*, *B. rapa*, *B. oleracea*, and *A. thaliana*, respectively (Table S1). The *A. thaliana* LOX genes identified in this study were identical to those described in a previous report [20], confirming the reliability of our results. The *RsLOX* genes were distributed on only five of the nine radish chromosomes (R01, R02, R05, R07, and R08). Additionally, R07 contained five LOX genes, which was the most of any of the radish chromosomes, followed by R01 and R02, both of which included two LOX genes (Figure 1).

**Figure 2:**
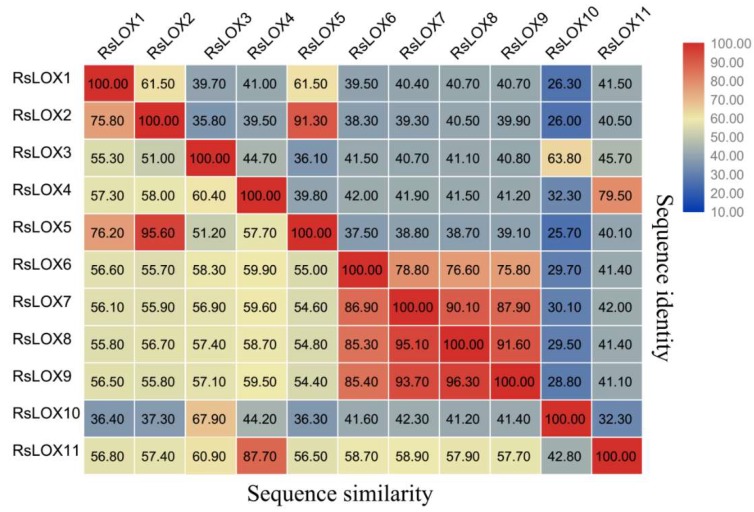
*R. sativus* LOX amino acid sequence identities and similarities (%).

The physical and chemical properties of all 11 *RsLOX* genes were analyzed (Table S2). The encoded RsLOX enzymes comprised 676 to 920 amino acids, with a predicted molecular weight of 77.01 to 103.32 kDa. Among the 11 RsLOXs, RsLOX10 was the shortest and had the lowest molecular weight. The predicted aromaticity ranged from 0.092 to 0.104, whereas the isoelectric point ranged from 5.17 to 9.11. These differences mostly result from variation in the nonconserved regions’ amino acid sequences. A comparison of the RsLOX sequences revealed a sequence identity of 18.7–91.3% and a sequence similarity of 27.4–95.6% at the amino acid level (Figure 2).

### 2.2. Phylogenetic Relationships among LOX Family Members from Diverse Plant Species

**Figure 3:**
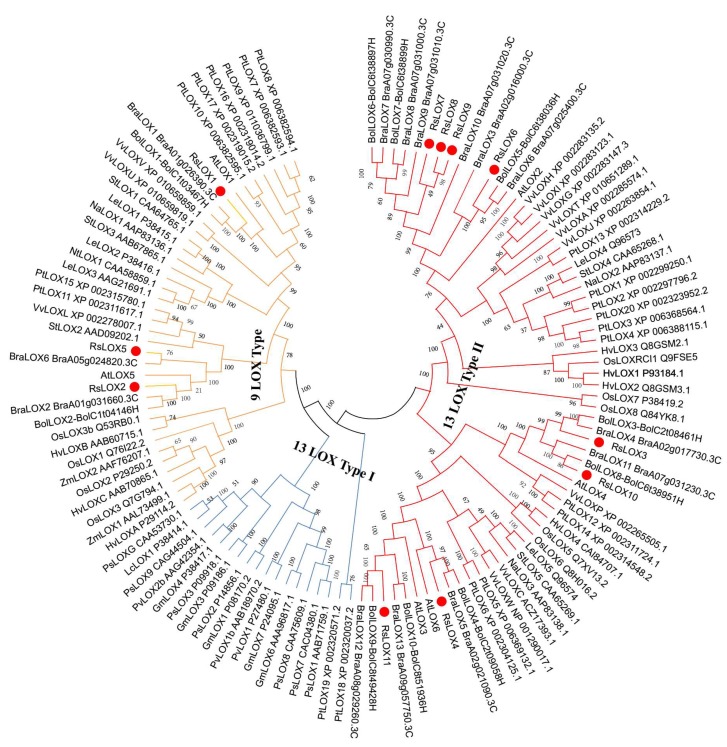
Phylogenetic relationships among LOXs from *R. sativus* and other species. The 9-LOX branches are presented in orange. The 13-LOX type I and type II branches are presented in blue and red, respectively. The identified radish LOXs are highlighted with a red circle. Species abbreviations are as provided in Table S3.

To determine the evolutionary relationships of the *R. sativus* LOX family members to other known LOX families, we constructed a phylogenetic tree based on 123 LOX amino acid sequences from 17 species (Figure 3 and Table S3). The tree categorized the LOXs as 9-LOX and 13-LOX enzymes. Of the 11 identified *R. sativus* LOXs, three (RsLOX1, RsLOX2, and RsLOX5) were characterized as 9-LOX enzymes, whereas the other eight RsLOXs were designated as 13-LOX enzymes. The RsLOXs were grouped with the LOXs from related species (i.e., *B. rapa* and *B. oleracea*). Furthermore, the 13-LOX enzymes were further classified as type I and II. However, the analyzed Brassica species lacked 13-LOX type I enzymes, which is consistent with previous report [36].

### 2.3. Conserved Domain and Structural Analyses of R. sativus LOXs

**Figure 4:**
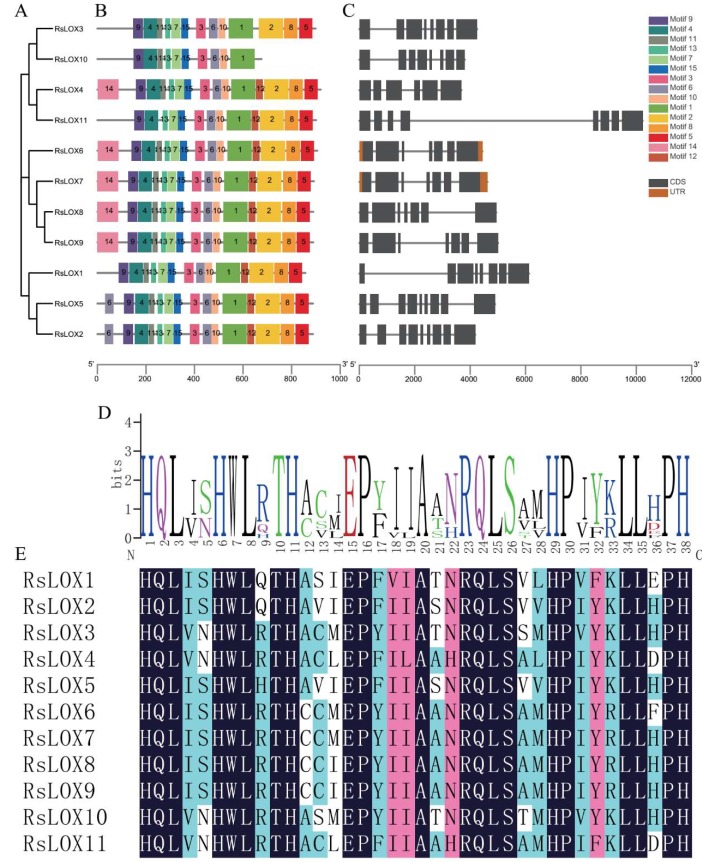
Phylogenetic, motif, structural, and conserved domain analyses of RsLOXs. (**A**) Phylogenetic tree comprising RsLOX proteins. (**B**) Schematic representation of the conserved RsLOX motifs. (**C**) Exon–intron structures in *R. sativus* LOX genes. (**D**) Sequences logo of a 38-residue RsLOX motif. (**E**) Alignment of a 38-residue conserved motif. The dark color indicates the amino acids totally matching, while the light indicates the part matching.

To further clarify the motifs and structural variations among RsLOXs, a separate phylogenetic tree with RsLOX protein sequences was constructed. Additionally, *RsLOX* exon–intron organizations and the encoded conserved motifs were compared. The phylogenetic relationships of the RsLOXs were identical to those of the RsLOXs in the phylogenetic tree based on sequences from 17 species (Figure 4A).

Most of the *RsLOX* genes shared similar exon–intron structures regarding the number and length of exons. The genes encoding 9-LOX enzymes (*RsLOX1*, *RsLOX2*, and *RsLOX5*) comprised seven to eight introns, whereas the genes for the 13-LOX enzymes had five to seven introns. Moreover, *RsLOX4* had the fewest introns (five), whereas RsLOX2 and RsLOX5 contained the most introns (eight) (Figure 4C).

To gain insight into potential functions and diversifcation of the *LOX* genes in *R. sativus*, we analyzed the predicted distinct motifs. As expected, most of closely related family members shared common motifs, suggesting they are functionally similar. Upon closer inspection, we determined that RsLOX10 lacks some of the conserved motifs present in other RsLOXs. Moreover, 12 of the 15 identified motifs (motifs 1–10, 12, and 15) were present in all analyzed LOXs (Figure 4B). Motif 1, which comprised 38 amino acid residues, was highly similar to the aforementioned LOX domain [His-(X)4-His-(X)4-His-(X)17-His-(X)8-His]. This motif was conserved in all 11 RsLOXs identified in this study, suggesting it may affect enzyme stability and activity (Figure 4D,E). An analysis of subcellular localization indicated that the 13-LOX type II enzymes included a chloroplast transit peptide, whereas RsLOX1, RsLOX2, and RsLOX5 (i.e., 9-LOX enzymes) did not (Table S4).

### 2.4. Tandem Duplications and Synteny of LOX Genes

**Figure 5:**
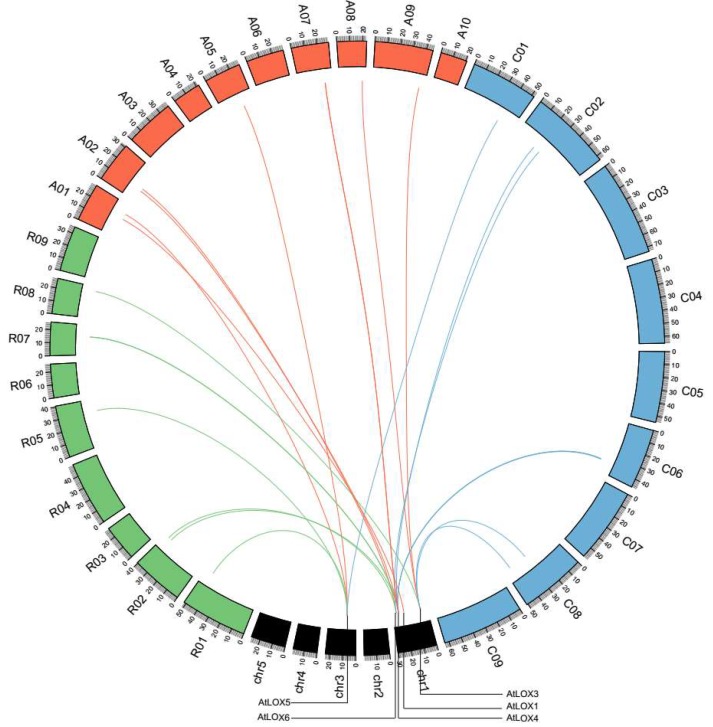
Syntenic relationships among *LOX* genes of *R. sativus*, *B. oleracea*, *B. rapa*, and *Arabidopsis* was visualized in a circos plot. The chromosomes of *Arabidopsis*, *R. sativus*, *B. rapa*, and *B. oleracea* were shaded with black, green, orange, and blue colors, respectively.

Whole genome and tandem duplications (TD) provide sources of primitive genetic material for genome complexity and evolutionary novelty [37]. We investigated the syntenic and tandem relationship of L*OX* genes between *A. thaliana* and *R. sativus* to trace the evolutionary history of L*OX* genes. Of the six *AtLOX* genes, four have a syntenic relationship with seven *RsLOX* genes. An analysis with the SynOrths program suggested all of these syntenic relationships formed via whole genome triplication (WGT) or segmental duplication (SD) events. Syntenic relationships were also detected among the genes of the other analyzed species (Table S5). The *AtLOX1* gene had a syntenic relationship with only one *B. rapa* gene. Additionally, *AtLOX2* lacked a syntenic gene in *R. sativus*, *B. oleracea*, and *B. rapa*. In contrast, *AtLOX3–6* had syntenic genes in all three of these species (Figure 5).

Interestingly, of the six *AtLOX* genes, *AtLOX4* had the most syntenic copies in *R. sativus*, *B. oleracea*, and *B. rapa.* Furthermore, the *AtLOX4* syntenic genes Rsa10033815, BraA07g030990.3C, and BolC6t38897H were tandemly duplicated. These observations implied that WGT and TD events substantially contributed to the expansion of the LOX gene families in *R. sativus*, *B. oleracea*, and *B. rapa* (Table S6).

### 2.5. Expression Profiles of R. sativus LOX Genes in Various Tissues

**Figure 6:**
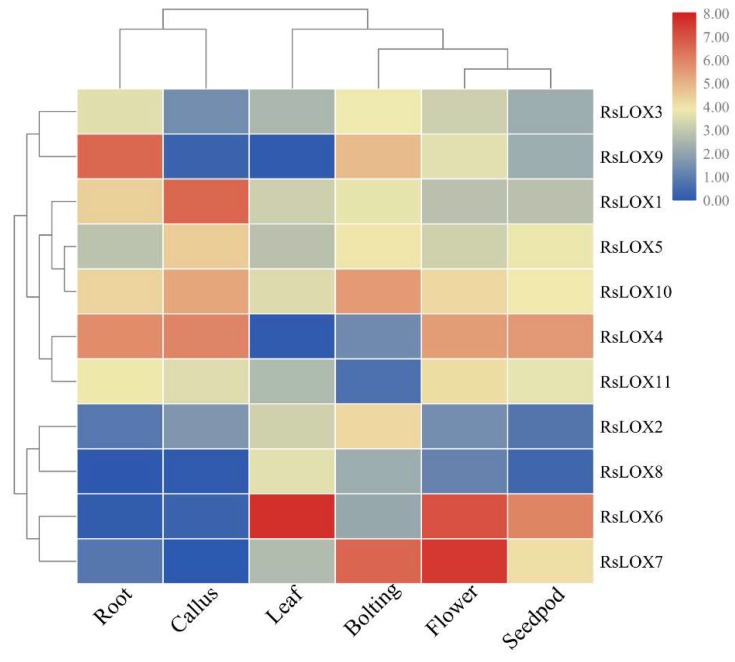
Hierarchical clustering of *R. sativus* LOX gene expression profiles in various tissues. The FPKM values were log-transformed and visualized in a heat map.

To clarify the *RsLOX* expression patterns, we analyzed previously reported transcriptome data for six tissues (flowers, siliques, leaves, stems, callus, and roots) (Table S7) [38]. A heat map representing *RsLOX* expression levels indicated these genes were expressed in several tissues, with some genes expressed mainly in particular tissues. A hierarchical cluster analysis classified the *RsLOX* genes into two main groups based on their expression patterns (Figure 6). However, these groups were not consistent with the 9-LOX and 13-LOX categorizations. The *RsLOX1* and *RsLOX2* genes, which encode 9-LOX enzymes, were similarly expressed and were clustered together, implying they may have similar functions. Duplicated genes may undergo a non-functionalization, neofunctionalization, or subfunctionalization [39]. The tandemly duplicated genes (*RsLOX7*, *RsLOX8*, and *RsLOX9*) were differentially expressed, implying they are functionally diverse.

### 2.6. Analysis of RsLOX Expression in Response to Abiotic and Biotic Stresses

**Figure 7:**
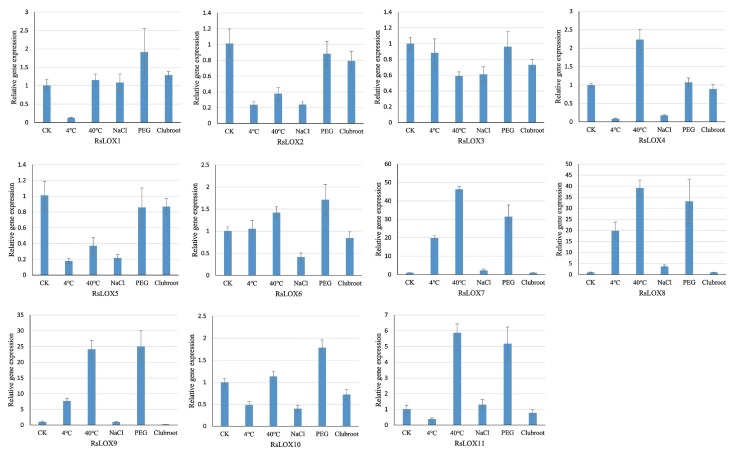
Quantitative real-time PCR analysis of *RsLOX* expression levels in the roots under various abiotic and biotic stress conditions. The presented gene expression levels are relative to the expression of the reference gene. Data are presented as the mean ± standard deviation of three independent experiments. CK: control treatment.

A quantitative real-time polymerase chain reaction (qRT-PCR) assay was performed to examine *RsLOX* expression levels in root tissue under high and low temperature, salinity, and simulated drought (polyethylene glycol (PEG) treatment) conditions (Figure 7). The qRT-PCR results revealed that the expression levels of the tandemly duplicated genes (*RsLOX7*, *RsLOX8*, and *RsLOX9*) were considerably upregulated by the high and low temperatures and the PEG treatment. Additionally, *RsLOX11* expression was upregulated approximately 5-fold in response to the high-temperature and PEG treatments.

Regarding the low-temperature treatment, the expression levels of all analyzed genes, except for *RsLOX7*, *RsLOX8*, and *RsLOX9*, were downregulated or unchanged. Similar to the effects of the low-temperature treatment, the exposure to NaCl increased the *RsLOX7* and *RsLOX8* expression levels by 2.2-fold and 3.7-fold, respectively, but decreased or did not affect the expression levels of the other *RsLOX* genes. Moreover, the expression levels of all *RsLOX* genes were not affected by the *Plasmodiophora brassicae* infection.

## 3. Discussion

With the increasing availability of genomic data, the LOX gene family has been studied in several plant species regarding their potential functions in development and stress responses. However, there has been relatively little research into the LOX gene families of *R. sativus* and Brassica species. We identified and characterized 11 *R. sativus* LOX genes in terms of their phylogenetic relationships, gene and protein structures, and expression profiles. We revealed diverse gene structures, conserved motifs, and differential expression patterns in various tissues and in response to abiotic stresses (cold, heat, simulated drought, and salinity). Our findings suggest the *RsLOX* genes have similar sequences and functions, but fulfill slightly different tissue-specific roles related to plant development and stress responses. Thus, the data presented herein provide insights into the *RsLOX* family and may be useful for functionally characterizing the *RsLOX* genes.

In this study, the *RsLOX* genes were divided into two groups (13-LOX and 9-LOX) according to phylogenetic relationships, which was consistent with the results of many previous studies [20,32,33,34,35]. Furthermore, the 9-LOX enzymes (RsLOX1, RsLOX2, and RsLOX5) have highly similar sequences (>75%), whereas, except for the enzymes encoded by the tandemly duplicated genes (*RsLOX7*, *RsLOX8*, and *RsLOX9*), the 13-LOX type II enzyme sequences are only moderately similar (Figure 1). Additionally, the 13-LOX type II RsLOXs contain a chloroplast transit peptide. Our findings are consistent with the results of previous studies that indicated the type I LOX sequences are highly similar and lack a transit peptide, whereas the type II LOX sequences are moderately similar and include an N-terminal chloroplast transit peptide [2,7]. Therefore, the classifications of the RsLOXs based on the phylogenetic tree and analyzed sequence characteristics were consistent, which confirmed the validity of our methodology and predictions. The analysis with the MEME suite and the examination of exon–intron structures revealed the similarity in the gene structures and motif compositions of the RsLOXs within sub-branches, suggesting these RsLOXs are functionally similar [40].

Gene duplication events (e.g., WGT, SD, and TD) have led to the expansion and increased functional diversity of specific gene families [41,42]. Previous studies concluded that a WGT event occurred in the common ancestor of *R. sativus*, *B. oleracea*, and *B. rapa* following its divergence from *A. thaliana* approximately 14–20 million years ago [43,44]. In the current study, we determined that four of the six *AtLOX* genes have syntenic copies in *R. sativus*, which is consistent with the evolution of these species. However, differences in the number of retained syntenic genes suggest there may have been some variability in the gene loss events during evolution. Functionally redundant genes are always lost in the diploidization process occurring after paleopolyploidy events [45]. In contrast, most of the *AtLOX4* syntenic copies, as well as the tandemly duplicated genes, are present in *R. sativus*, *B. oleracea*, and *B. rapa*, suggesting the associated tandem duplication occurred in the common ancestor of these three species. The gene dosage effects hypothesis suggests that the increase in the *AtLOX4* copy number reflects the considerable need for the encoded enzyme in multiple complex physiological and biological processes [46]. Our findings may be useful for elucidating the expansion of the *LOX* gene family.

Gene expression patterns can provide important clues regarding gene functions. Previous research produced evidence that LOXs play fundamental roles in plant development and stress responses. In the current study, *RsLOX* expression patterns were observed that reproductive organs were clustered together. Under stress conditions, the expression levels of the 9-LOX genes (*RsLOX1*, *RsLOX2*, and *RsLOX5*) were downregulated, which is similar to pepper 9-LOX gene expression patterns in response to JA and thrips [47]. These results are consistent with the fact that 9-LOX enzymes mediate insect resistance, protein storage, and tuber development [2,18]. The 13-LOX enzymes reportedly contribute to abiotic stress responses [16,17]. The data presented herein indicated that the expression levels of *RsLOX7*, *RsLOX8*, *RsLOX9*, and *RsLOX11* (i.e., 13-LOX genes) are considerably affected by several stresses. Moreover, RsLOX7, RsLOX8, and RsLOX9 clustered with AtLOX2 in the phylogenetic tree. In *A. thaliana*, AtLOX2 is required for wound-induced JA accumulation, but whether it is involved in responses to other stresses remains unclear [23]. JA is important for regulating plant responses to various abiotic stresses, including cold and drought conditions [48,49,50]. Therefore, it is reasonable to speculate that *RsLOX7*, *RsLOX8*, and *RsLOX9* are the main *RsLOXs* in the JA biosynthetic pathway, and the increased production of JA is directly involved in responses to various environmental stresses. We also observed that the *RsLOXs* are unaffected by NaCl and *P. brassicae* treatments, possibly because of an insufficient processing time or concentration. Alternatively, the *RsLOXs* may simply be unresponsive to these two stresses. The predicted *RsLOX* functions will need to be experimentally verified in future studies.

## 4. Materials and Methods

### 4.1. Sequence Acquisition and Identification of LOX Genes

The *A. thaliana* genome assemblies were downloaded from the TAIR10 database [51]. Genome resources for *R. sativus* [52] and *B. rapa* [53] were downloaded from the BRAD database (http://brassicadb.org/brad/) [54], and *B. oleracea* HDEM genome data [55] was download from Genoscope database. The LOX domain model (PF00305) was downloaded from the Pfam database 32.0 [56]. Additionally, Hmmsearch, from the HMMER suite (version 3.1) [57], was used to search for the LOX domain (PF00305) in the proteins of each species. The putative LOX proteins among the initially screened proteins were filtered with an E-value cutoff of 1 × 10^−5^ and sequence coverage of the Pfam domain models of at least 60%. The detected proteins were then examined for the presence of the LOX and PLAT domains with the InterProScan website. The Biopython module Bio.SeqUtils.ProtParam was used to predict the molecular weight, isoelectric point, and other physical and chemical properties of the *R. sativus* LOX proteins. The MG2C website was used to visualize the location of *RsLOX* genes on nine pseudomolecular chromosomes [58].

### 4.2. Multiple Sequence Alignment and Phylogenetic Tree Construction

The global alignment tool Needle of the EMBOSS software suite [59] was used for the pairwise alignment of RsLOX proteins to determine the sequence identity and similarity. The LOX proteins from 17 species were included in a phylogenetic analysis. The amino acid sequences for the *R. sativus*, *B. oleracea*, *B. rapa*, and *A. thaliana* LOX proteins were identified in this study, whereas the sequences for the other 13 species were obtained from published articles [36,60]. The MUSCLE program [61] was used to align the complete LOX amino acid sequences. A phylogenetic tree was constructed with the MEGA-X program [62]. Specifically, the neighbor-joining method with the Jones–Taylor–Thornton model was used. The phylogenetic tree was constructed with 500 bootstrap replicates used to assess the statistical support for each tree node. Additionally, uniform rates and homogeneous lineages were applied and a partial deletion with a site coverage cutoff of 70% was used for treating gaps. Another phylogenetic tree comprising RsLOX proteins was constructed using the same methods.

### 4.3. Analyses of Conserved Motifs, Gene Structures, and Subcellular Localizations

To identify the conserved motifs encoded by LOX genes, the following parameters of the MEME suite [63] were applied to search for motifs: the maximum number to be found was set to 15 and the motif window length was set to 8–100 bp. The TBtools program [64] was used to analyze gene structures and for visualizing gene structures and the encoded conserved domains. Sequence logos for the conserved LOX domain in *R. sativus* proteins were generated with WebLogo 3 [65]. Additionally, the conserved 38-amino acid sequences were aligned with the DNAMAN program (version 9) (Lynnon Biosoft Company, Quebec, QC, Canada). The subcellular localizations of RsLOXs were predicted with the online ChloroP 1.1 Server [66].

### 4.4. Analyses of Tandem Duplications and Synteny

To clarify the evolution of the LOX genes in *R*. *sativus* and related species, we analyzed the tandem duplications and syntenic relationships of LOX genes in *R*. *sativus*, *A*. *thaliana*, *B. oleracea*, and *B. rapa*. Tandem duplications were defined as gene copies that were separated by 10 or fewer genes and within 200 kb. The SynOrths program was used to identify syntenic orthologs based on the sequence similarity and the collinearity of the flanking genes [67], and the circos software was used to visualize the syntenic relationships of LOXs among these species [68].

### 4.5. Transcriptional Profile Analysis

The *RsLOX* expression profiles were analyzed based on published RNA sequencing data for six *R. sativus* tissues (flowers, siliques, leaves, stems, callus, and roots) [38]. The reads were aligned to the *R. sativus* “XYB-36-2” genome [52] with TopHat2 [69]. The transcript abundance for each gene was calculated according to the fragments per kilobase of transcript per million mapped reads (FPKM) values with the Cufflinks program [70].

### 4.6. Plant Materials and Stress Treatments

Seeds of radish variety ‘BXC02′ were incubated at 25 °C for 2 days in darkness. Germinated seeds were sown in plastic pots and incubated in a growth chamber with a 16-h day (24 °C)/8-h night (21 °C) cycle. Seedlings at the six true-leaf stage were used for the subsequent experiments. For the heat and cold stress treatments, the seedlings were incubated at 40 °C and 6 °C for 24 h, respectively. The control seedlings were subjected to a 24 °C (day)/21 °C (night) cycle. For the simulated drought and salinity stress treatments, the seedlings were subjected to 20% PEG 6000 and 300 mM NaCl, respectively, for 24 h with a 24 °C (day)/21 °C (night) cycle. Regarding the *P. brassicae* infection, a 5-mL aliquot of a 3 × 10^8^/mL resting spore suspension was injected in the soil near the roots of seedlings at the four-leaf stage. The roots were harvested and analyzed 7 days after the inoculation. The control seedlings were treated with sterile water. The taproots of five seedlings were collected as an independent biological replicate, and each treatment was completed with three replicates. The samples were stored at −80 °C prior to the subsequent RNA isolation step.

### 4.7. RNA Extraction and qRT-PCR Analysis

Total RNA was extracted from all samples with the Trizol reagent (Invitrogen, Carlsbad, CA, USA). A DNase I treatment was used to eliminate any contaminating genomic DNA. The quality of the RNA samples was checked with the NanoDrop 2000 spectrophotometer (ThermoFisher Scientific, Beijing, China) and 1% denaturing agarose gels. The RNA was used as the template for the first-strand cDNA synthesis with PrimeScript reverse transcriptase (TaKaRa Biotechnology, Dalian, China). The Primer Premier 5.0 program was used to design gene-specific primers (Table S8). Additionally, the *RsGAPDH* gene was used as an internal control for normalizing the gene expression data [71]. The *RsLOX* expression levels were analyzed in a qRT-PCR assay, which was completed with the SYBR Green qPCR kit (TaKaRa Biotechnology, Dalian, China) and the Stratagene Mx3000P thermocycler (Agilent, Santa Clara, CA, USA). The PCR program was as follows: 95 °C for 5 min then 40 cycles of 95 °C for 15 s and 60 °C for 30 s. The relative LOX gene expression levels were calculated with the 2^−ΔΔ*C*t^ method [72]. The analysis included three biological replicates, each with three technical replicates.

## 5. Conclusions

In this study, we comprehensively characterized the radish LOX gene family via a systematic approach comprising analyses of phylogeny, gene structure, motif composition, evolution, and gene expression patterns in response to various abiotic and biotic stresses. Our results revealed the conservation and diversity in the sequence characteristics and functions among *RsLOX* genes. Moreover, a qRT-PCR analysis of *RsLOX* expression indicated that three tandem-clustered *RsLOX* genes are involved in responses to various environmental stresses through the jasmonic acid pathway. This comprehensive analysis provides a foundation for the functional characterization of LOX genes under stress conditions in radish and potentially in other species, including cotton and other Brassicaceae species.

## Supplementary Materials

Supplementary materials can be found at https://www.mdpi.com/1422-0067/20/23/6095/s1. Table S1: Protein LOX and their alignment information in radish and related species. Table S2: Characteristics of protein RsLOX. Table S3: Plant LOX protein sequences used for phylogenetic tree construction. Table S4: Subcellular localization analysis of radish LOXs. Table S5: Syntenic relationships of LOX between *A. thaliana* and radish, *B. oleracea*, *B. rapa*. Table S6: Tandem duplicated LOX genes in radish, *B. oleracea* and *B. rapa*. Table S7: FPKM of LOX genes in different radish tissues. Table S8: RT-PCR primers of *RsLOXs*.

## Abbreviations

LOXs	Lipoxygenases
PLAT	Polycystin-1, lipoxygenase, alpha-toxin
His	Histidine
9-HPOD	9s-hydroperoxyoctadecadienoic acid
13-HPOD	13s-hydroperoxyoctadecadienoic acid
JA	Jasmonic acid
WGT	Genome triplication
SD	Segmental duplication
TD	Tandem duplication
PEG	Polyethylene glycol
qRT-PCR	Quantitative real-time polymerase chain reaction
FPKM	Fragments per kilobase of transcript per million mapped reads
